# An efficient density peak cluster algorithm for improving policy evaluation performance

**DOI:** 10.1038/s41598-022-08637-8

**Published:** 2022-03-23

**Authors:** Zhenhua Yu, Yanghao Yan, Fan Deng, Fei Zhang, Zhiwu Li

**Affiliations:** 1 Institute of Systems Security and Control, College of Computer Science and Technology, Xi’an University of Science and Technology, Xi’an, 710054 China; 2 Hitachi Building Technology (Guangzhou) Co., Ltd, Guangzhou, 510700 China; 3 Institute of Systems Engineering, Macau University of Science and Technology, Taipa, Macau China

**Keywords:** Computational science, Computer science, Information technology, Software

## Abstract

In recent years, the XACML (eXtensible Access Control Markup Language) is widely used in a variety of research fields, especially in access control. However, when policy sets defined by the XACML become large and complex, the policy evaluation time increases significantly. In order to improve policy evaluation performance, we propose an optimization algorithm based on the DPCA (Density Peak Cluster Algorithm) to improve the clustering effect on large-scale complex policy sets. Combined with this algorithm, an efficient policy evaluation engine, named DPEngine, is proposed to speed up policy matching and reduce the policy evaluation time. We compare the policy evaluation time of DPEngine with the Sun PDP, HPEngine, XEngine and SBA-XACML. The experiment results show that (1) when the number of requests reaches 10,000, the DPEngine evaluation time on a large-scale policy set with 100,000 rules is approximately 2.23%, 3.47%, 3.67% and 4.06% of that of the Sun PDP, HPEngine, XEngine and SBA-XACML, respectively and (2) as the number of requests increases, the DPEngine evaluation time grows linearly. Compared with other policy evaluation engines, the DPEngine has the advantages of efficiency and stability.

## Introduction

At present, access control has become not only an important research object in fields of network and information security but also a research hot spot in interdisciplinary subjects of Internet of things^1^, Industry 4.0^2^, CPS (Cyber-Physical System)^3^, Block-chain^4^, Cloud computing^5^ and big data^6^. With continuous growths of network and information system security requirements, access control has become a key to promoting the rapid development of critical industries.

In the research of access control, policies are used more and more frequently to describe the security requirements of network and information authorization service systems. The access control is an important part of the network security requirement module in authorization service systems. It means that when users access authorized network service systems, the systems control or protect the access to existing resources through the identity authentication, dynamic authorization and other methods. Recently, the XACML^7^ (eXtensible Access Control Markup Language) is widely used to define architectures of access control mechanisms and express access control policies. First, in an XACML access control model^8^, users send requests to a PEP (Policy Enforcement Point), which dispatches requests to a PDP (Policy Decision Point). Then, the PDP queries the request attributes from a PIP (Policy Information Point) and traverses policies in a PAP (Policy Administrator Point). Finally, the PDP makes evaluation decisions and returns them to the PEP as responses. Obviously, the PDP is one of the most important modules in an XACML access control model. The PDP evaluation performance is crucial for an authorization service system^10^. However, as interactions between users and servers increase, the size and complexity of policy sets increase correspondingly^11^. As a result, it becomes more difficult and time-consuming to evaluate a large number of requests.

In order to improve the PDP evaluation performance, there have been many relevant research achievements in recent years, including distributed authorization systems, decision graphs, eliminations of conflicts and redundancies, etc. However, the PDP evaluation performance is still restricted by the following multiple factors.

1. Both distributed authorization systems and decision graphs need to spend much time evaluating requests for dealing with large-scale policy sets.
2. The elimination of conflicts and redundancies is beneficial to optimizing policy sets, but the improvement of the PDP evaluation performance is restrained.
3. It is extremely difficult to construct decision graphs for large-scale policy sets. Although decision graphs consume a lot of storage space, the PDP evaluation performance is still limited.

Therefore, how to improve the PDP evaluation performance is a challenge for large-scale policy sets. Existing evaluation methods are inefficient in dealing with large-scale and complex policy sets. Using clustering algorithms to optimize policy sets is effective for improving policy evaluation performance. Many powerful and novel clustering algorithms are proposed to solve many different problems. Hassan et al.^12^ propose a meta-heuristic clustering algorithm, which can process heterogeneous data sets with multiple features. In Ref.^13^, an adaptive evolutionary clustering algorithm is developed to solve the Formal Context's ambiguity problem. Mohammed et al.^14^ propose a meta-heuristic algorithm by simulating the reproductive behavior of bee colonies, which is applied to classic pressure vessel design problems and achieves good results. Askari^15^ modifies the Fuzzy C-Means algorithm to be suitable for data with unequal cluster sizes, noise and outliers, and uneven distribution of quality. However, clustering algorithms have different adaptability to data sets of different shapes and sizes, and it is difficult to achieve excellent effects on large-scale and complex policy sets. In order to greatly improve the evaluation efficiency of PDP, it is urgent to propose a clustering algorithm that can effectively deal with large-scale policy sets. To solve these problems, we propose an improved clustering algorithm and construct an efficient policy evaluation engine. Our contributions are described as follows.

1. In order to better deal with large-scale complex policy sets, an optimization algorithm based on the DPCA (Density Peak Cluster Algorithm)^16^ is proposed, which can realize clustering analyses of arbitrary shapes and multi-dimensional policy sets and automatically determine parameters to improve the clustering performance for policy sets.
2. In the policy matching stage, policy sets are processed by labeling according to clustering results to form a new policy tag set. The tag set can significantly speed up policy matching and save storage space.
3. A policy evaluation engine based on the optimization algorithm, named DPEngine, is constructed and has an excellent evaluation performance for large-scale complex policy sets.

The rest of this paper is organized as follows. In section “Related works”, some related works are reviewed. Section “Preliminary” introduces the details of XACML and policy evaluation. Section “Approach overview” provides a brief introduction to the proposed policy evaluation engine, named DPEngine. In section “An effective policy evaluation engine”, we discuss an improved clustering algorithm and the details of the DPEngine. Section “Experimental results and analyses” shows experiment results and analyses. Finally, the paper is summarized in section “Conclusions”.

## Related works

In the past few years, most researchers have made great contributions to improve the PDP evaluation performance. These contributions are mainly divided into the following aspects.

### Distributed authorization systems

Compared with traditional centralized authorization models, distributed authorization models can handle plenty of requests more efficiently. Wang et al.^17^ analyze topological characteristics of different policies and apply a greedy algorithm to divide a policy set into multiple subsets, making them suitable for distributed systems. Daniel et al.^18^ redefine a default XACML architecture, such that the PAP can effectively manage policy sets belonging to the XACML architecture. Deng et al.^19^ make a policy decomposition technology based on an ant colony algorithm, which decomposes a policy set into multiple sub-policies, so as to reduce the cost of policy deployment. Lischka et al.^20^ put forward a distributed method, which analyzes the architecture of XACML policies and makes authorization decisions according to the attributes of policy sets.

### Elimination of conflicts and redundancies

Jebbaoui et al.^21^ develop a new method based on sets and semantics. First, they design a set with an intermediate representation to reduce the complexity of policies. Second, the meanings of policy rules are analyzed through a structural inference of rules and semantic verification of deductive logic, detecting defects, conflicts and redundancies in rules. The method provides an accurate and effective analysis of XACML policies. Wang et al.^22^ present a new policy evaluation engine based on a multi-level optimization technology. The policy evaluation engine adopts a multi-cache mechanism to eliminate redundant rules. Ngo et al.^23^ address an XACML model based on decision graphs. Logical expressions in policies are transformed into a decision tree structure. The XACML model can effectively detect conflicts and redundancy in policies. Shaikh et al.^24^ introduce a new technique to detect inconsistent policies. They put forward an improved algorithm to analyze and construct a decision tree that includes a Boolean expression normalization policy set, and then an anomaly detection algorithm is executed to secure a final detection result.

Although the elimination of conflicts and redundancies is beneficial to improving the PDP evaluation efficiency, existing research efforts still lack better solutions for dealing with large policy sets.

### Decision graphs

Liu et al.^25^ develop a policy evaluation engine called XEngine, which advances a fast policy evaluation algorithm to speed up processing requests. Ros et al.^26^ raise a new policy evaluation model based on a decision tree structure. The model can not only search applicable rules quickly but also evaluate requests more efficiently, thus further improving the policy evaluation efficiency. Deng et al.^27^ introduce an efficient policy evaluation engine combined with an automaton theory. The engine establishes an attribute bitmap for each policy and makes evaluation results quickly through the attribute bitmaps. Turkmen et al.^28^ come up with a policy evaluation method using the SMT (SAT modulo theories). The method not only improves the policy evaluation efficiency but also can be applied to a wide range of other fields.

Decision graphs can effectively speed up the policy evaluation efficiency. However, if the size of a policy set is extraordinarily large, its decision tree will be difficult to build.

### Other methods

Marouf et al.^29^ propose an adaptive optimization method for XACML policies. They process policy sets with the *k*-means algorithm and dynamically optimize ranking access control policies, thus improving the policy evaluation efficiency. Mourad et al.^30^ construct a framework, named SBA-XACML, which is based on a set language. The SBA-XACML framework converts an XACML structure into a readable mathematical grammar. The framework not only improves the policy evaluation efficiency but also allows the detection of conflicts and redundancies. Deng et al.^31^ address an optimized XACML policy management scheme based on bitmap storage and HashMap. A policy set is digitized, and then a sequential storage structure based on an array is established, such that rules can be indexed quickly. Cheminod et al.^32^ present a comprehensive access control policy refinement and validation method, which can detect errors in the policy execution in time and perform policy matching correctly. Rezvani et al.^33^ use ASP (Answer Set Programming) to analyze various characteristics of an XACML policy including redundancies and conflicts, thus improving the policy evaluation efficiency.

With the rapid development and progress of computer technologies, the scale and complexity of policy sets in an authorization service system are increasing. The existing research has been unable to meet the application requirements for large-scale policy sets. This calls for an efficient and stable policy evaluation engine to deal with large-scale policy sets.

## Preliminary

Before describing the proposed evaluation engine in detail, this section introduces the concept of XACML and the problem description of policy evaluation.

### Concepts of XACML

In XACML, a policy set contains thousands of policies, and each policy has many rules. A rule in a policy set can be expressed in Eq. (1).

1 $$Rule = \left\langle {Subject,\;Resource,\;Action,\;Condition,\;Effect} \right\rangle$$

where the *Subject* is request proposer, the *Resource* usually contains data, files, and URL. The *Action* typically includes reading and writing. The *Condition* represents constraints. The *Effect* has two values: Permit and Deny. The *Effect* is the core attribute of a rule. It is used to decide whether to accept the current request and perform the corresponding operation. Accordingly, a request can be expressed as

2 $${\text{Request}} = \left\langle {Subject,\;Resource,\;Action,\;Condition} \right\rangle$$

where attributes contained in a request are consistent with those in a rule.

### Problem description of policy evaluation

Policy evaluation is a process of matching attributes in a request with attributes of rules in a policy set. The attributes to be matched include *Subject*, *Resource*, *Action* and *Condition*. If attributes in a request are different from those in all rules of a policy set, users will be denied access. On the contrary, the corresponding operation is performed according to the value of *Effect* in the rule. In an authorization service system, policy sets of the access control are very large and complex. Each request needs many matches to find the corresponding policy to perform correct operations. At the same time, the number of requests is also very large, and multitudes of requests also cause a great burden to the system. Therefore, in a large-scale authorization service system, deciding how to improve the efficiency of policy evaluation is of great significance to the access control ability of the whole system.

## Approach overview

**Figure 1:**
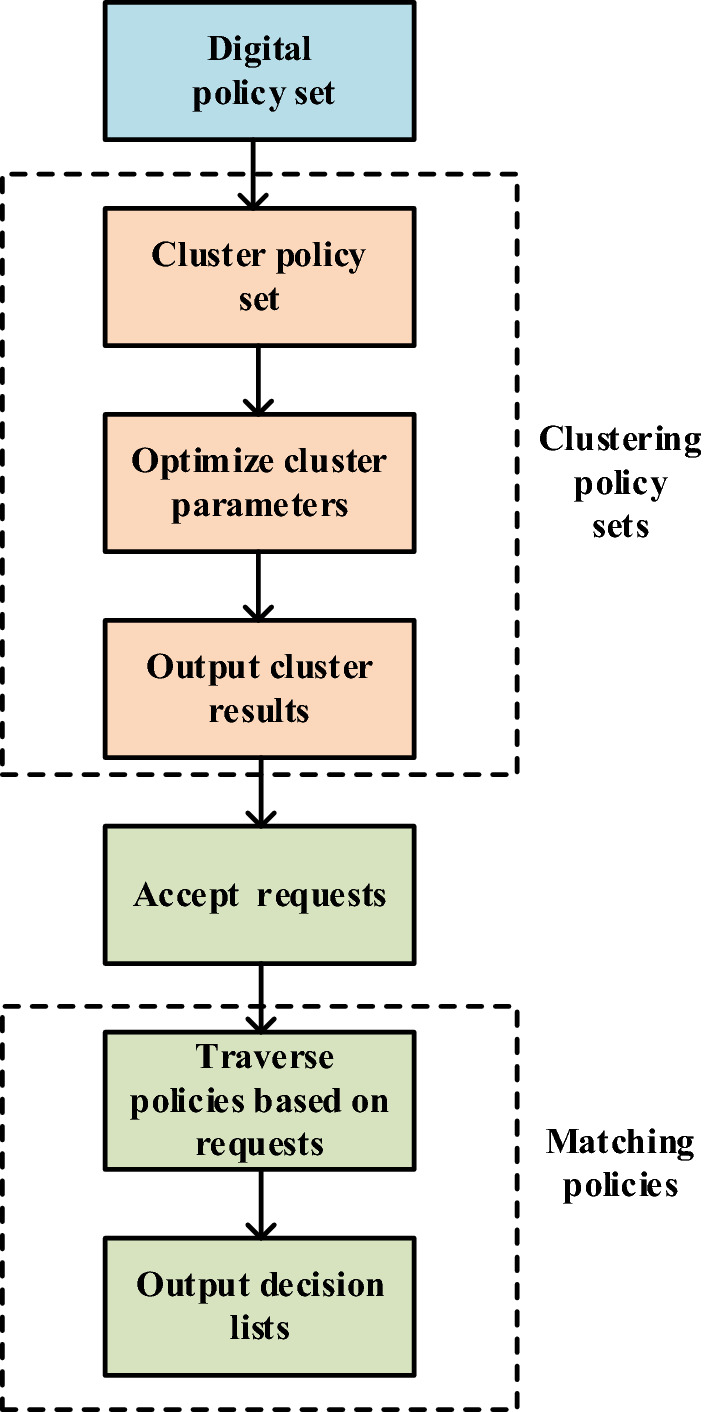
Working process of DPEngine.

Based on an optimization algorithm, a policy evaluation Engine, named DPEngine, is conducted to improve the PDP evaluation performance. The DPEngine can load policies, evaluate requests and return evaluation decisions to the PEP. The DPEngine has three main functions, namely, preprocessing policy sets, clustering policy sets, and matching policies. Its working process is shown in Fig. 1.

In the preprocessing part of policy sets, the DPEngine can digitize attributes of each policy in policy sets. Each policy in a policy set has five attributes: *Subject*, *Resource*, *Action*, *Condition*, and *Effect*. These attributes are translated into digital representations by a random sort. The result is a complete digitized policy set, which is beneficial to the clustering and matching parts later. The details of preprocessing are covered in section “Preprocess for policy sets”.

**Figure 2:**
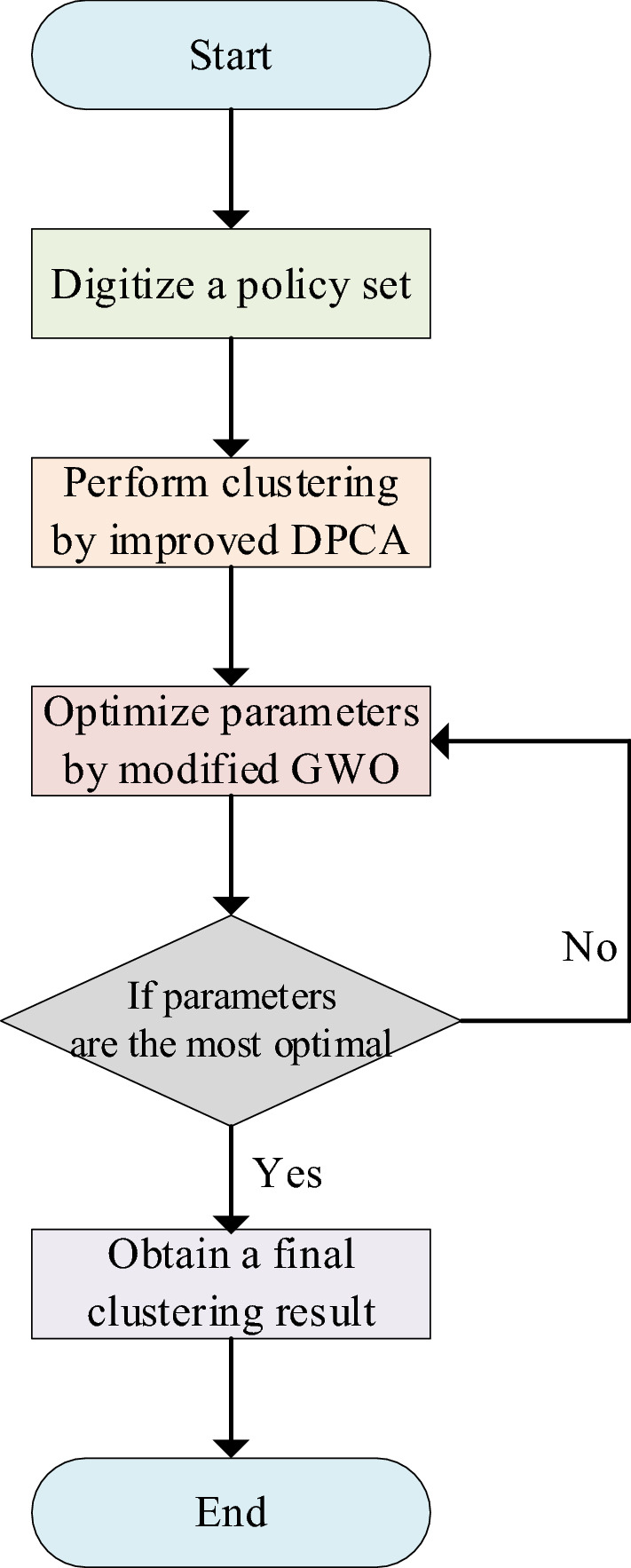
Clustering process of an improved DPCA.

After the preprocessing part is finished, the second part is to cluster policy sets. The core of DPEngine is to obtain the best cluster results by adopting an optimization algorithm. In order to improve clustering effects, a modified version of the GWO (Grey Wolf Optimizer)^34^ is used to optimize parameters in the optimization algorithm. Then, according to the obtained optimal parameters, we perform clustering to secure final clustering results. The whole clustering process is shown in Fig. 2.

The third part is the policy matching. Based on clustering results, similar policies are grouped together and labeled with the same tags. When a request arrives, its corresponding label can be determined based on its attributes. By quickly retrieving policies with the same tag, a match between policies and requests can be completed. According to the match result, an evaluation decision can be made. The evaluation decision will be returned to the PEP to help a service system make an authorization decision. The details of policy matching are introduced in section “Matching policies”.

## An effective policy evaluation engine

In order to improve the policy evaluation performance on large-scale complex policy sets, we construct an efficient policy evaluation engine, called DPEngine. The engine can preprocess policy sets, use an optimization algorithm to efficiently and reasonably cluster policy sets, and make fast policy matching according to the clustering results. This section covers the details of the DPEngine.

### Preprocess for policy sets

**Figure 3:**
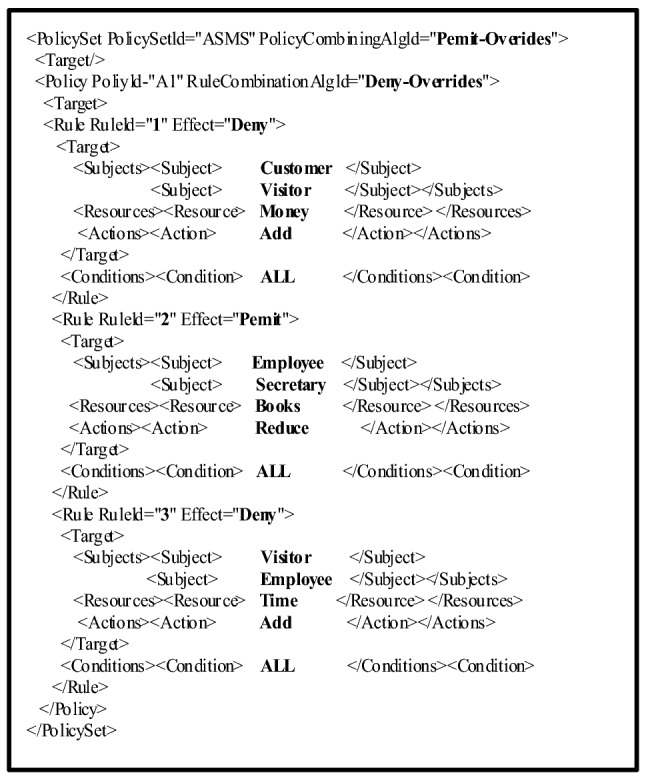
A simple example of an XACML policy set.

In order to meet the needs of the algorithm, a policy set should be preprocessed. An initial policy is shown in Fig. 3.

In Fig. 3, each policy has five attributes: *Subject*, *Resource*, *Action*, *Condition*, and *Effect*. Each attribute is represented as a consecutive integer starting from 0, based on a random sorting result in a policy set. The *Effect* is represented by 0 or 1. The processed policy set is shown in Table 1.

**Table 1:** A processed policy set.

No.	Subject	Resource	Action	condition	Effect
0	0	0	0	0	0
1	1	1	1	0	1
2	2	2	0	0	1

### Clustering for policy sets

In order to perform effective and reasonable clustering on large-scale complex policy sets, we present an optimization algorithm based on the DPCA (Density Peak Clustering Algorithm)^12^ and GWO (Grey Wolf Optimizer)^34^, which are explained in detail below.

#### Density peak clustering algorithm

The Density Peak Clustering Algorithm is a classic density clustering algorithm, which can effectively cluster arbitrary shape data sets. This is appropriate for dealing with complex policy sets. According to the preprocessing results of policy sets, each policy is transformed into a data point that has five attributes: *Subject*, *Resource*, *Action*, *Condition*, and *Effect*, thus forming a new policy point set. The algorithm mainly has two assumptions for policy sets:

1. The number of policy points within a certain range around a policy point is called local density. The local density of a core policy point is large enough, and the local density of its surrounding policy points is not higher than it.
2. The distance between a core policy point and other core policy points with high local densities is far enough.

Based on the two assumptions, the local density of each policy point *i* can be expressed as

3 $$\rho_{i} = \sum\limits_{j \ne i} {\chi (dist_{ij} - dist_{c} )}$$

where *dist*_*ij*_ denotes the Euclidean distance between policy point *i* and policy point *j*, and *dist*_*c*_ represents the cut-off distance. The function $\chi (x)$ can be expressed as

4 $$\chi (x) = \left\{ {\begin{array}{*{20}l} {1,} & {\quad x < 0} \\ {0,} & {\quad x \ge 0} \\ \end{array} } \right.$$

According to Eqs. (3) and (4), the number of policy points whose distance from policy point *i* is less than the cut-off distance is taken as the local density of the policy point *i*.

The key lies in the selection of *δ*_*i*_-cluster center distance that is used to measure the distance between different core policy points. According to the definition of local density, we can calculate the local density of each policy point *i*, and determine the cluster center distance *δ*_*i*_ according to the density. The densities of all policy points are sorted from large to small. If the policy point *i* is a point with the highest density, its cluster center distance *δ*_*i*_ is equal to the distance from the farthest policy point *j* to it, which can be expressed as

5 $$\delta_{i} = \mathop {\max }\limits_{j} (dist_{ij} )$$

If a policy point *i* is not a point with the highest density, its cluster center distance is equal to the distance with the nearest policy point *j* whose density is bigger than the point, which can be expressed as

6 $$\delta_{i} = \mathop {\min }\limits_{{j:\rho_{j} > \rho_{i} }} (dist_{ij} )$$

**Figure 4:**
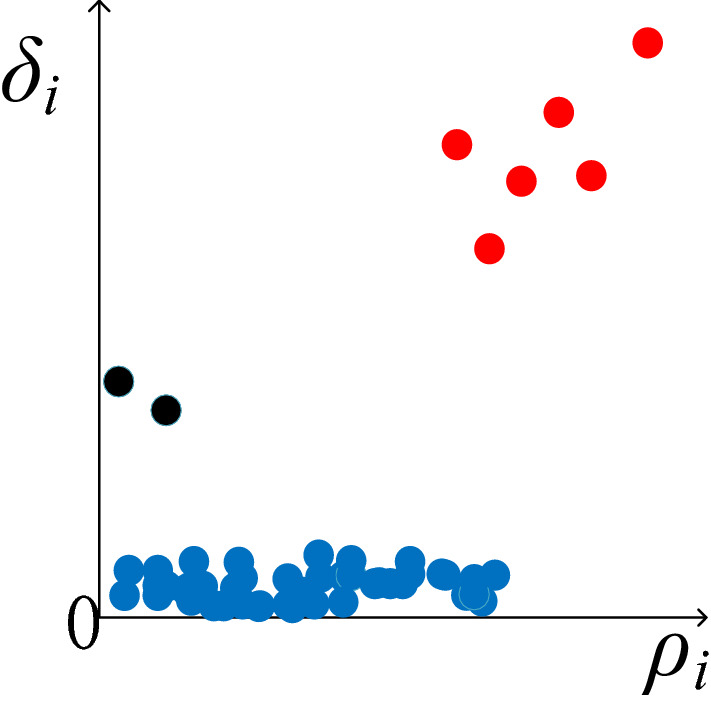
*ρ*_*i*_*–δ*_*i*_ decision graph.

As demonstrated by Hassan and Rashid^12^, after the cluster center distances and local densities of all policy points are calculated, the result is represented as a two-dimensional decision graph in Fig. 4 by plotting their values according to *δ*_*i*_ and *ρ*_*i*_ as the coordinate axes.

As can be seen from Fig. 4, a core policy point is far away from the coordinate axis, while a normal policy point is near the horizontal axis, and an abnormal policy point is near the vertical axis. In the case of small policy sets, core policy points can be easily obtained by observations. A quantitative analysis can be done with Eq. (5).

7 $$\gamma_{i} = \rho_{i} \times \delta_{i}$$

**Figure 5:**
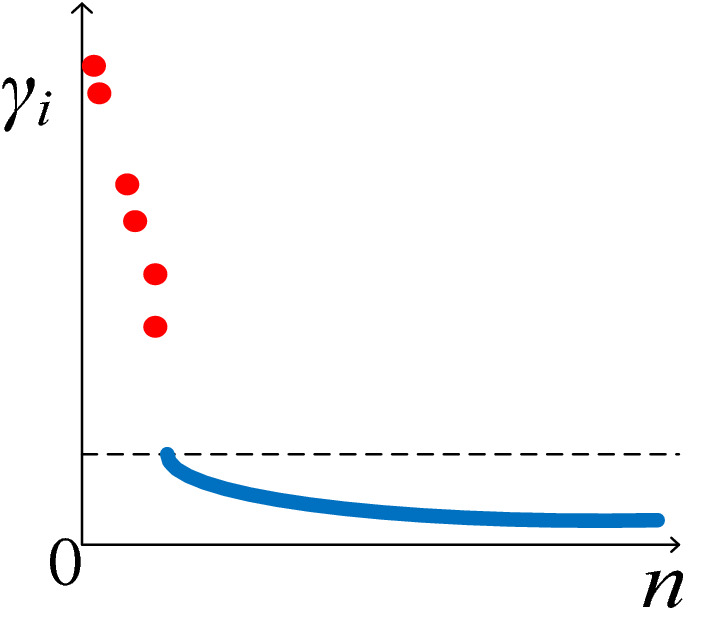
Value of *γ*_*i*_ in descending order.

According to Eq. (5), the values of *γ*_*i*_ for all policy points are calculated, and then they are ranked from largest to smallest. The larger the *γ*_*i*_ of a policy point is, the more likely it is to be a core policy point. By taking *γ*_*i*_ as a vertical axis and policy point *i* as a horizontal axis, a two-dimensional plane is drawn, as shown in Fig. 5.

As can be seen from Fig. 5, the curve of normal policy points is relatively smooth, while that of core policy points is extremely steep. There is a clear gap between the two types of policy points. It is easy to distinguish a core policy point from a normal policy point. Therefore, the DPCA can realize a clustering analysis of multi-dimensional policy sets by constructing two-dimensional plane graphs.

However, the number of core policy points is difficult to observe for a large-scale policy set. This shortcoming restricts the cluster performance. At the same time, due to the limitation of clustering performance, further improvement of policy evaluation efficiency is also constrained. Therefore, in order to achieve a better cluster effect, we make an optimization method that adopts the modified GWO^34^ to optimize its parameters. The main optimization target is to acquire the optimal number of core policy points.

#### A modified grey wolf optimizer

In recent years, a large number of swarm intelligence optimization algorithms are applied to various disciplines and fields. The GWO (Grey Wolf Optimizer)^34^ is a classic swarm intelligent optimization algorithm which is an optimized search method inspired by the preying activities of grey wolves and has the advantages of strong convergence, fewer parameters and easy implementation. Rashid et al.^35^ propose an improved version of the GWO, which is used to optimize parameters of the RNN to improve classification accuracy. Mohammed et al.^36^ use the *k*-means algorithm to enhance the limitations of grey wolves attack and search process. Rashid et al.^37^ improve the performance of GWO to find the optimal solution by embedding the search phase of GWO into the development phase of WOA (Whale optimization algorithm)^38^. Khandelwal et al.^39^ develop a modified GWO to solve the TNEP (transmission network expansion planning) problem. With the ideas of Rashid et al., we also propose a modified version to improve the optimization performance of the GWO. The details of our algorithm improvements are described below.

The GWO seeks the best solution to problems by simulating hunting behaviors of grey wolves. When grey wolves search for their prey, they gradually approach and surround it. A mathematical model simulating the behavior is as follows.

8 $$\overrightarrow {D} = \left| {\overrightarrow {C} \cdot \overrightarrow {{X_{p} }} (t) - \overrightarrow {X} (t)} \right|$$

9 $$\overrightarrow {X} (t + 1) = \overrightarrow {X}_{p} (t) - \overrightarrow {A} \cdot \overrightarrow {D}$$

10 $$\overrightarrow {A} = 2\overrightarrow {h} \cdot \overrightarrow {{r_{1} }} - \overrightarrow {h}$$

11 $$\overrightarrow {C} = 2\overrightarrow {{r_{2} }}$$

where *t* is the number of current iterations, $\vec{A}$ and $\vec{C}$ are synergistic coefficient vectors, X_p_(t) represents a position vector of the prey, X(*t*) represents a position vector of the current grey wolves, *h* decreases linearly from 2 to 0 during a whole iteration, and *r*_1_/*r*_2_ is a random vector in [0, 1].

In order to simulate the search behaviors of grey wolves, it is assumed that wolves *a*, *b*, and *c* have a strong ability to identify potential prey locations. Therefore, during each iteration, the best three wolves *a*, *b* and *c* in the current population are retained, and the positions of other search agents are updated based on their location information. A mathematical model simulating the behavior can be expressed as follows.

12 $$\overrightarrow {D}_{a} = \left| {\overrightarrow {C}_{1} \cdot \overrightarrow {X}_{a} - \overrightarrow {X} } \right|$$

13 $$\overrightarrow {D}_{b} = \left| {\overrightarrow {C}_{2} \cdot \overrightarrow {X}_{b} - \overrightarrow {X} } \right|$$

14 $$\overrightarrow {D}_{c} = \left| {\overrightarrow {C}_{3} \cdot \overrightarrow {X}_{c} - \overrightarrow {X} } \right|$$

15 $$\overrightarrow {X}_{1} = \overrightarrow {X}_{a} - \overrightarrow {A}_{1} \cdot \overrightarrow {D}_{a}$$

16 $$\overrightarrow {X}_{2} = \overrightarrow {X}_{b} - \overrightarrow {A}_{2} \cdot \overrightarrow {D}_{b}$$

17 $$\overrightarrow {X}_{3} = \overrightarrow {X}_{c} - \overrightarrow {A}_{3} \cdot \overrightarrow {D}_{c}$$

18 $$\overrightarrow {X} (t + 1) = \frac{{\overrightarrow {X}_{1} + \overrightarrow {X}_{2} + \overrightarrow {X}_{3} }}{3}$$

where $\vec{X}a$, $\vec{X}b$, and $\vec{X}c$ respectively represent position vectors of wolves *a*, *b*, and *c* in the current population; $\vec{X}$ represents a wolf position vector; $\vec{D}a$, $\vec{D}b$, and $\vec{D}c$ respectively represent the distances between the current candidate wolf and the three optimal wolves.

In the original algorithm, there are three optimal wolves to help find the best solution. However, the optimal solution may fall in the remaining locations. Therefore, our improvement is to add another wolf to participate in location updates, called wolf *d.* In the proposed improved GWO, the position of wolf *d* is also updated according to the positions of wolves *a*, *b*, and *c*. The following position-updated is used for the wolf *d*:

19 $$\overrightarrow {D}_{d} = \frac{1}{3}\left( {\overrightarrow {D}_{a} + \overrightarrow {D}_{b} + \overrightarrow {D}_{c} } \right)$$

20 $$\overrightarrow {X}_{4} = \overrightarrow {X}_{d} - \overrightarrow {A}_{4} \cdot \overrightarrow {D}_{d}$$

Since the wolf *d* is added as another optimal solution, Accordingly, Eq. (18) is updated as follows.

21 $$\overrightarrow {X} (t + 1) = \frac{1}{4}\sum\limits_{i = 1}^{4} {\overrightarrow {X}_{i} }$$

**Figure 6:**
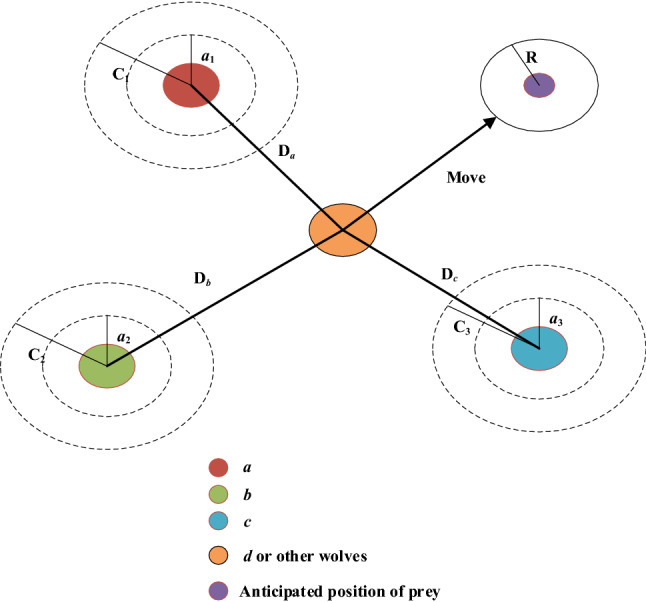
Position updating of wolves.

A wolf position updating process is shown in Fig. 6. The position of a candidate solution eventually falls into the random position defined by wolves *a*, *b*, *c* and *d*. Grey wolves mostly search for their prey according to the position of *a*, *b*, *c,* and *d*. In summary, the algorithm creates a random grey wolf population. During iterations, the leading wolves estimate the most likely prey location. Each candidate solution updates its distance from the prey. Finally, the algorithm is terminated after the specific situation is satisfied. The details of the modified Grey Wolf Optimizer are given in Algorithm 1.
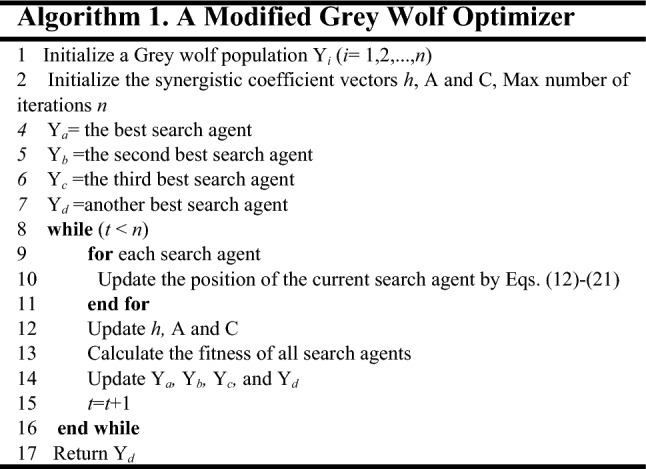

#### An optimization algorithm for clustering large-scale policy sets

In order to deal with large-scale policy sets, we propose an optimization algorithm. The modified GWO is adopted to optimize the number of core policy points. In the optimization process, the Silhouette index^40^ is taken as an objective function. The number of core policy points will be adjusted repeatedly until the clustering effect reaches the optimal.

At present, there are many clustering evaluation indexes. The Silhouette index is an internal evaluation index, which can evaluate the quality of clustering by the number of clusters. The Silhouette index is calculated according to Eq. (22).

22 $$Sil(i) = \frac{[b(i) - a(i)]}{{\max [a(i),\;b(i)]}}$$

where *a*(*i*) is an average distance between the policy point *i* and other policy points in the same cluster. The smaller *a*(*i*) is, the higher the probability that the policy point *i* belongs to the cluster is. *b*(*i*) represents the minimum value of the average distance between policy point *i* and all policy points in other clusters. The larger *b*(*i*) is, the lower the probability that the policy point *i* belongs to other clusters is. Obviously, *Sil*(*i*) is a value in [− 1, 1]. *Sil*(*i*) is close to 1, which indicates that the clustering of policy point *i* is reasonable. If *Sil*(*i*) is close to − 1, it indicates that policy point *i* should be classified into another cluster. If *Sil*(*i*) is approximately equal to 0, it means that the policy point *i* is on the boundary of two clusters. By calculating the Silhouette index of different cluster numbers, we can obtain clustering results that maximize the Silhouette index. In the proposed optimization algorithm, the number of core policy points keeps updating until it finds the global optimal number of core policy points.

After the number of core policy points is obtained, the corresponding number of policy points is selected as core policy points according to their local density and cluster center distance. These core policy points are used as clustering centers to perform clustering and acquire the final clustering results. The specific process of the optimization algorithm is detailed in Algorithm 2.
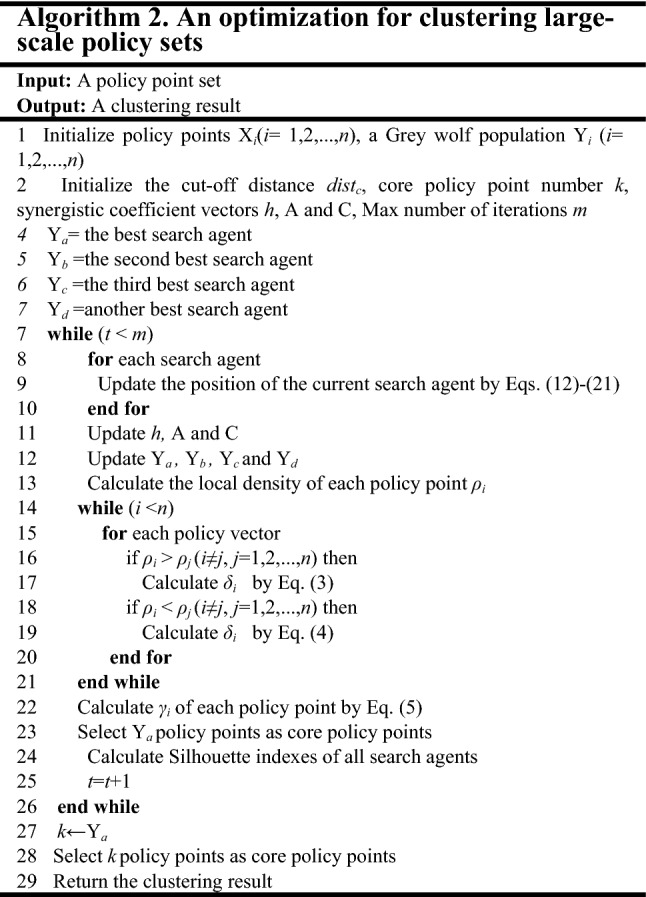


In Algorithm 1, from line 1 to line 6, a policy point set, a grey wolf population and related parameters are initialized. The initial optimal solution is calculated. From line 8 to line 16, through continuous iterative updating, the optimal number of core policy points is obtained. From line 17 to line 29, the corresponding core policy points are selected as clustering centers, and the final clustering result is obtained. In Algorithm 1, the number of policy points is *n* and the max number of iterations is *m.* The time complexity of the algorithm is O(*mn*). The proposed optimization algorithm provides a more accurate method to determine the number of core policy points for large-scale policy sets. Therefore, its clustering effect for complex large-scale policy sets is further improved.

### Matching policies

**Figure 7:**
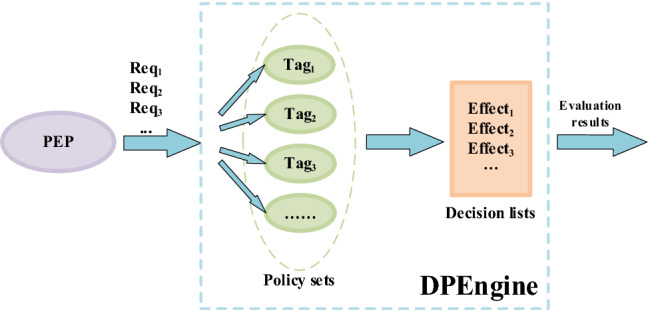
Process of policy matching.

The DPEngine obtains clustering results in policy sets by using the proposed optimization algorithm. Based on the clustering results, similar policies are grouped and labeled with the same tags to form a new policy tag set. When requests from the PEP arrive, their corresponding tags are determined by verifying the attributes of requests. By the new policy tag set, a quick retrieval of policies with the same tags can be achieved. Once the retrieval is completed, one can find policies that match the attributes of requests. According to the value of *Effect* in policies, the corresponding decision list is generated and the appropriate authorization decisions can be made. The specific process of policy matching is shown in Fig. 7.

## Experimental results and analyses

In this part, the DPEngine is compared with several existing policy evaluation engines, and experimental results are analyzed and discussed. The implementation of DPEngine is based on Python 3.8. The experiments are made on a laptop with a Windows operating system, which has 16 GB of memory and 1.80 GHz AMD Ryzen-7 4800U processors.

In order to verify that the DPEngine can efficiently evaluate complex and large-scale policy sets, we make three experiments as follows.

1. On three different policy sets, an optimization algorithm is used to cluster policies. In order to achieve the best cluster effect, the optimization algorithm adopts the modified GWO algorithm to optimize its parameter. The main optimization parameter is the core policy points number.
2. We compare and analyze the policy evaluation time of five policy evaluation engines: the DPEngine, Sun PDP^41^, HPEngine^42^, XEngine^25^ and SBA-XACML^30^, on three policy sets of different sizes and complexities.
3. We compare evaluation time experiments of the DPEngine on three policy sets of different sizes and complexities.

The Sun PDP is the first open-source XACML evaluation engine. It has been widely used and is an accepted industry standard. The HPEngine adopts a statistical analysis mechanism to reduce policy size and optimize matching methods. The XEngine is a policy evaluation engine that first transforms an XACML policy into a numeric policy. The SBA-XACML contains formal semantics and algorithms that use mathematical operations to improve policy evaluation performance.

### Experimental policy sets

In order to acquire authentic and credible experimental results, we simulate practical application scenarios and select three XACML access control policy sets widely used as follows.

1. Auction Management System (ASMS)^43^: A policy set of an access control rights management system. It primarily serves an auction process and restricts access to each user.
2. Virtual Management System (VMS)^44^: A policy set related to a virtual meeting management system. It is mainly used to control access rights of administrators, supervisors, and employees, etc.
3. Library Management System (LMS)^45^: A policy set for a library access service. Users include administrators, teachers, students, etc.

According to the experiment needs, we expand the number of rules for the ASMS, LMS, and VMS to 10,000, 50,000 and 100,000, respectively. We conduct experiments on the three policy sets of different sizes and complexities.

### Parameter optimization experiments

**Figure 8:**
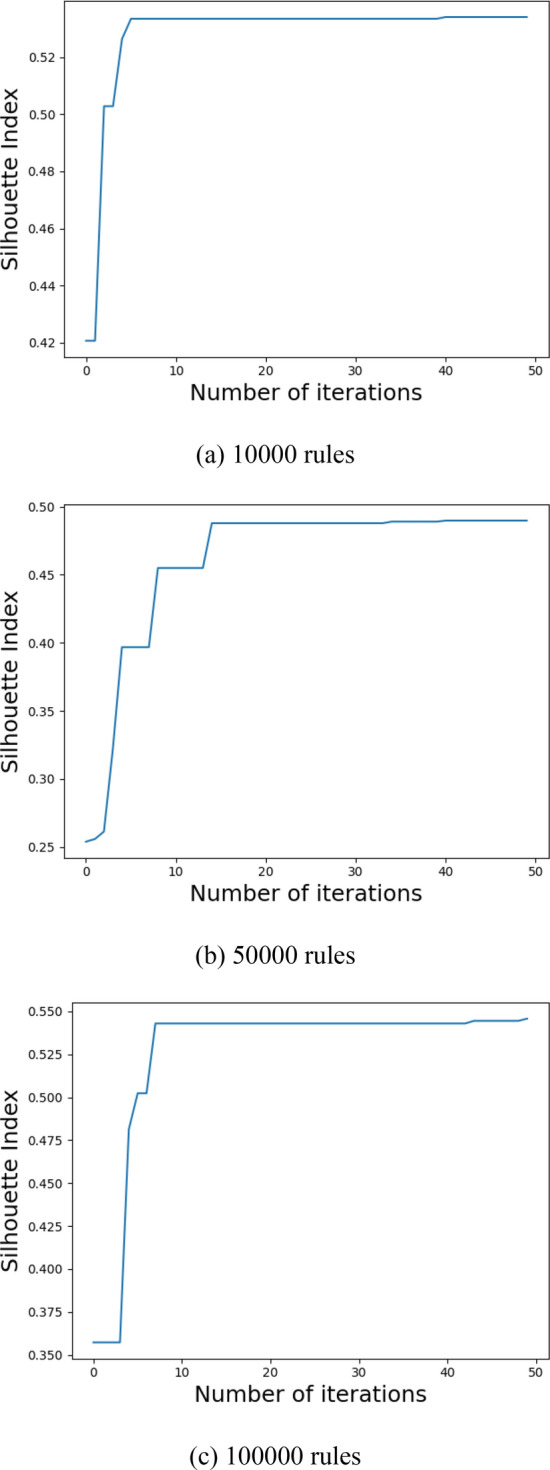
Parameter optimization on ASMS policy set.

**Figure 9:**
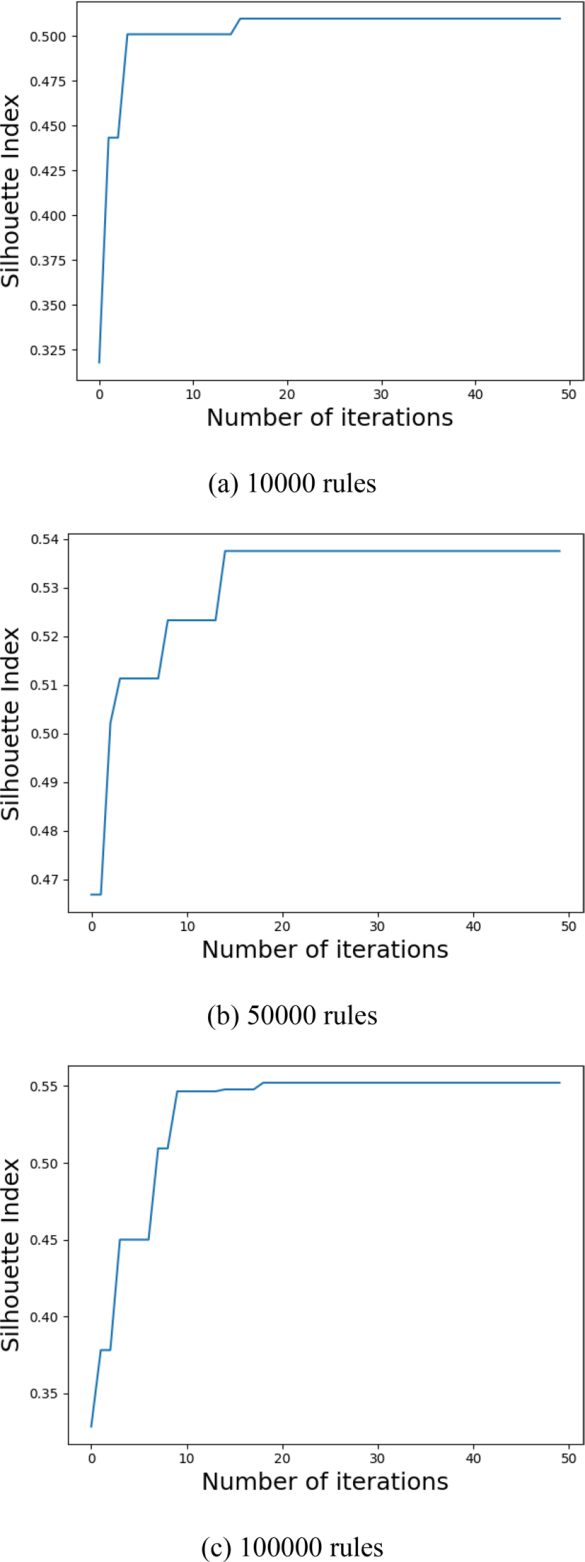
Parameter optimization on LMS policy set.

**Figure 10:**
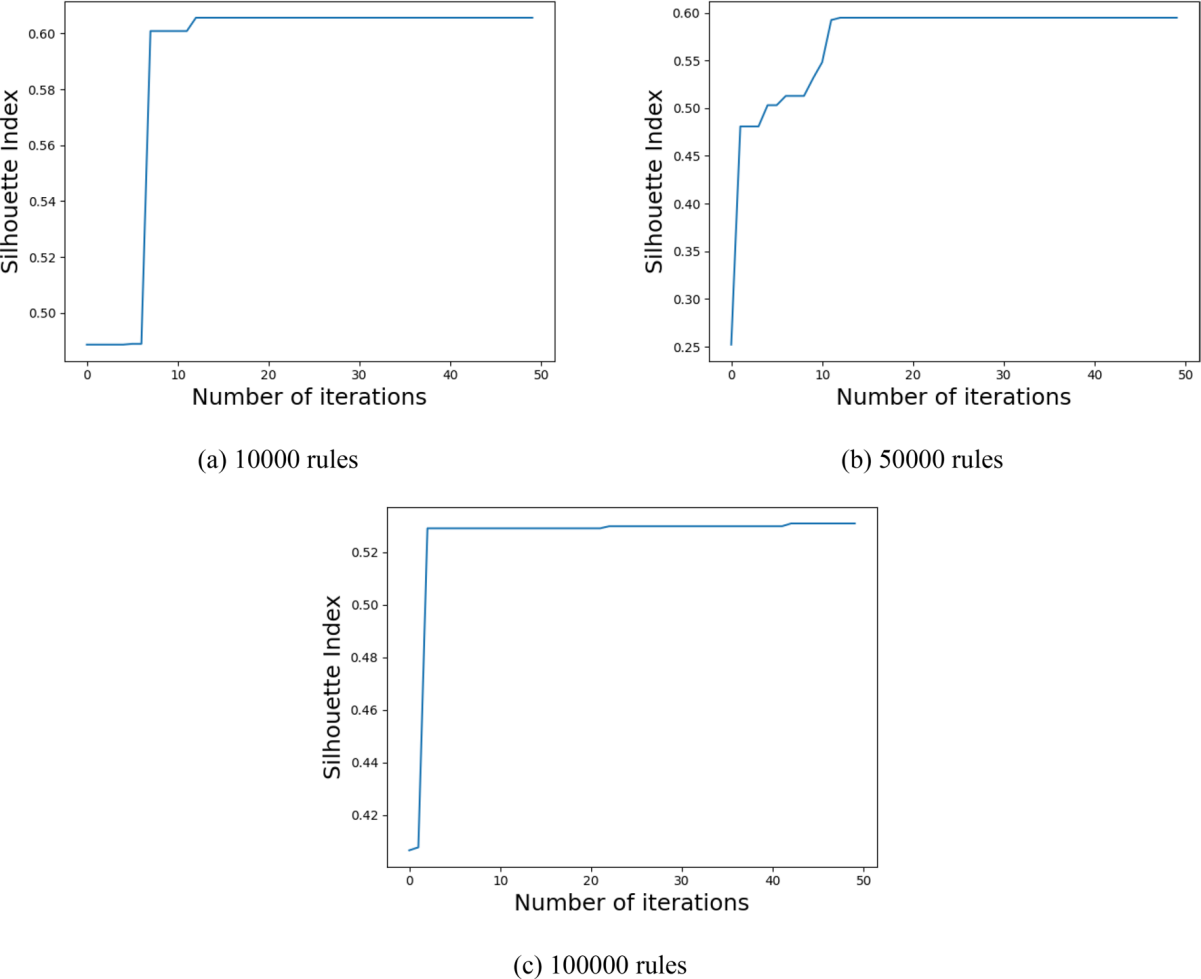
Parameter optimization on VMS policy set.

Before making the formal policy evaluation experiment, to achieve the best clustering accuracy, we use the modified GWO to optimize the core policy point number *k*. Experiments are conducted for the ASMS, LMS, VMS with 10,000, 50,000, and 100,000 rules, as shown in Figs. 8, 9, 10. In the optimization process, we choose the Silhouette index as an objective function. Using the modified GWO algorithm, the number of core policy points is continuously optimized through multiple clustering. By multiple clustering, the value of the Silhouette index changes with the number of core policy points. Figures 8, 9 and 10**,** respectively**,** represent the process of obtaining the value of the maximum Silhouette index through continuous optimization using the proposed optimization algorithm on ASMS, LMS and VMS policy sets of different scales. When the value of the Silhouette index reaches the maximum and tends to be stable, it indicates that the number *k* of core policy points has reached the best.

After the optimal number *k* of core policy points is obtained on the three policy sets, *k* core policy points are selected as cluster centers to gain the optimal cluster results. The clustering effect has a crucial impact on the speed of policy evaluation. The better clustering effects are of great help to the subsequent policy evaluation experiments.

### Comparisons of evaluating performance

**Figure 11:**
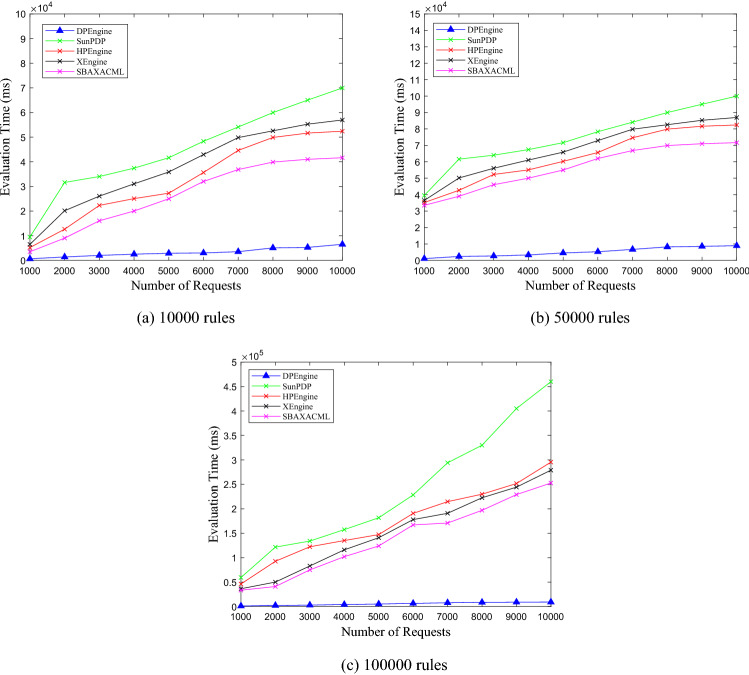
Evaluation performance on ASMS policy set.

**Figure 12:**
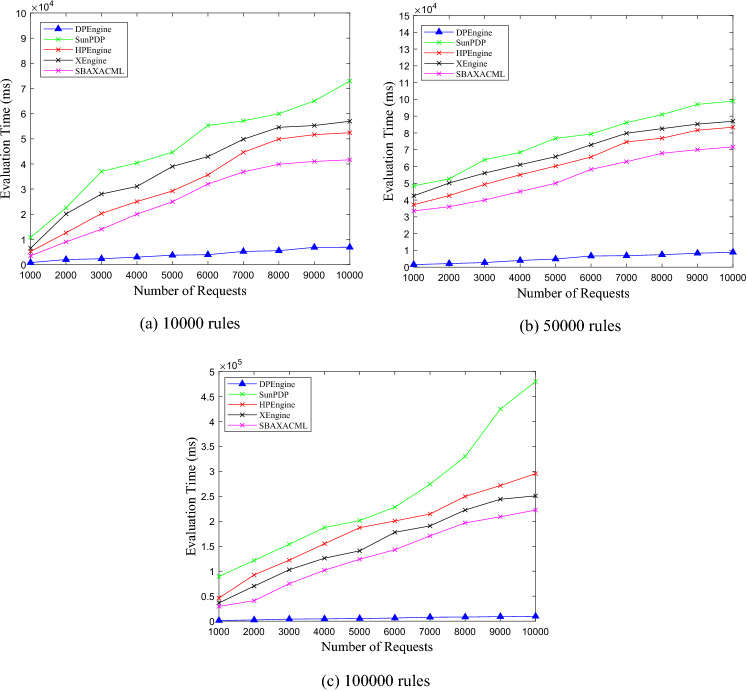
Evaluation performance on LMS policy set.

**Figure 13:**
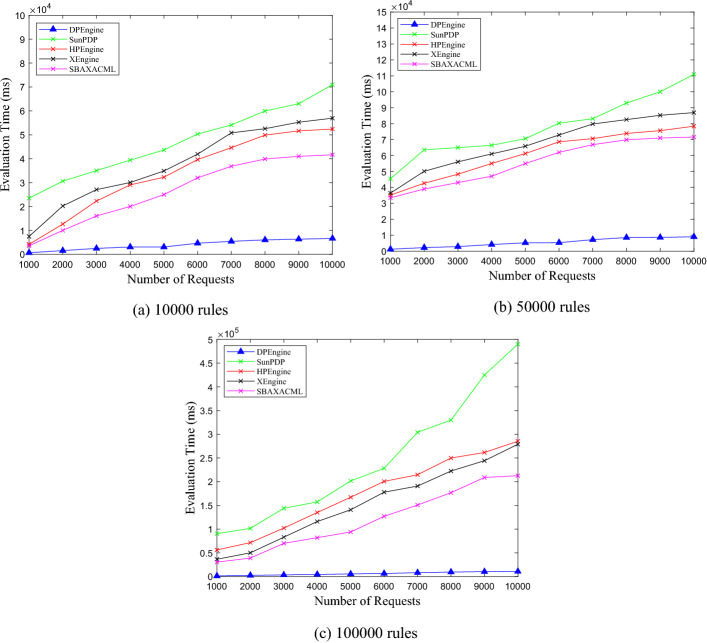
Evaluation performance on VMS policy set.

After achieving the best clustering effect, for the ASMS, LMS, VMS with 10,000, 50,000, and 100,000 rules, we test the evaluation time of DPEngine, Sun PDP, HPEngine, XEngine and SBA-XACML. In the experiment, the number of requests ranges from 1000 to 10,000, with an interval of 1000. The experimental results are shown in Figs. 11, 12, 13.

From Figs. 11, 12, 13, we can see that

1. for the three policy sets with 10,000, 50,000 and 100,000 rules respectively, the average evaluation time of DPEngine is much less than that of the Sun PDP, HPEngine, XEngine and SBA-XACML, respectively.
2. as the number of requests increases, the evaluation time of the Sun PDP, HPEngine, XEngine and SBA-XACML increases rapidly. However, the growth rate of the DPEngine evaluation time is much slower than that of the four evaluation engines above, and there is no significant increase.
3. as the scales of the three policy sets grow, the evaluation time curves of DPEngine have stable shapes without significant fluctuations, and the evaluation time for processing a single request is always controlled within approximately 0.6 ms. Thus, the DPEngine has great stability for large-scale policy sets.

### Comparisons of DPEngine on different policy sets

**Figure 14:**
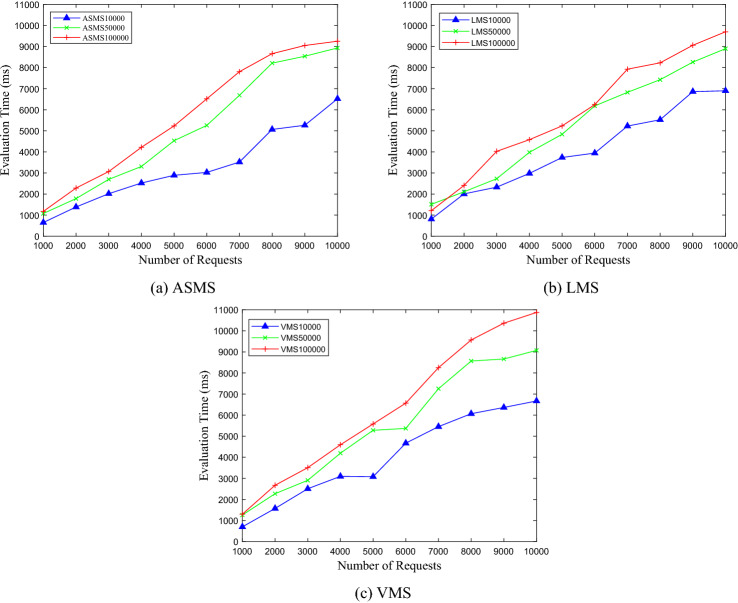
Comparison on different sizes policy sets.

The experiments are conducted on three policy sets with different sizes. We randomly generate 1000, 2000, …, 10,000 access requests, and record the evaluation time of DPEngine. For different sizes of policy sets with 10,000, 50,000, and 100,000 rules, the variations of evaluation time with the number of requests are shown in Fig. 14.

From Fig. 14, we conclude that

1. for the three policy sets with 10,000, 50,000 and 100,000 rules respectively, the evaluation time of DPEngine increases linearly as the requests increase without an obvious explosive growth.
2. for the three policy sets with 10,000, 50,000 and 100,000 rules respectively, the evaluation time of DPEngine has not increased significantly as the sizes of policy sets increase.

**Figure 15:**
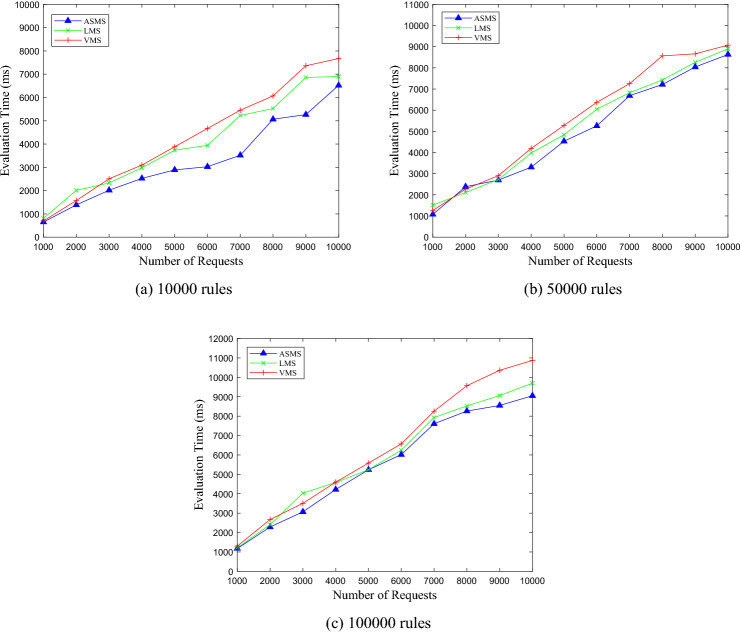
Comparison on different policy sets.

In order to further verify the performance of DPEngine, we conduct comparative experiments on the three policy sets with 10,000, 50,000, and 100,000 rules, respectively, which are shown in Fig. 15.

From Fig. 15, we generalize that

1. since the complexity of policies in the LMS and VMS is higher than that of the ASMS, the evaluation time of DPEngine spent on the LMS and VMS is longer.
2. the evaluation time curves of DPEngine on policy sets of different complexity increase linearly and slowly without obvious fluctuations, indicating the stability of DPEngine on different policy sets.

## Conclusions

In order to improve the PDP evaluation performance, a policy evaluation engine named DPEngine is proposed in this paper. The DPEngine divides all rules in a policy set into different categories by an optimization algorithm based on the DPCA, and gains labels corresponding to each category. When a request arrives, the DPEngine matches it with a corresponding subject. The DPEngine runs a parallel search in the tags corresponding to the subject to quickly match specific rules. The experimental results show that the DPEngine has significantly improved the PDP evaluation performance of large-scale policy sets compared with the Sun PDP, HPEngine, XEngine, and SBA-XACML. It has better adaptability to complex policy sets. Although the proposed optimization algorithm has excellent cluster performance on XACML policy sets, the space complexity of this algorithm can be further improved. At the same time, some new and more powerful algorithms can further improve the PDP evaluation efficiency. Nowadays, deep learning has been applied in many different disciplines and achieved many amazing results. The success of deep learning also has a lot of reference significance for our research. The idea of adopting some emerging deep models may further improve the evaluation efficiency of PDP. In the future, we plan to explore better methods and continue to improve the PDP evaluation performance. The proposed method can be applied to access control of social networks^46^ and parameter estimation of COVID-19 dynamical model^47,48^.

## Data Availability

The data and code that support the findings of this study are available from the corresponding author upon reasonable request.
